# Supplementary material for: Changes in the Microbial Composition of the Cecum and Histomorphometric Analysis of Its Epithelium in Broilers Fed with Feed Mixture Containing Fermented Rapeseed Meal
Source: Microorganisms. 2021 Feb 12;9(2):360. doi: 10.3390/microorganisms9020360 (PMC7918838; doi:10.3390/microorganisms9020360)
Supplement: Supplementary file 1 [file microorganisms-09-00360-s001.pdf]

Supplementary Material

Microorganism detection by chromogenic agar media and identification by MALDI-TOF-MS

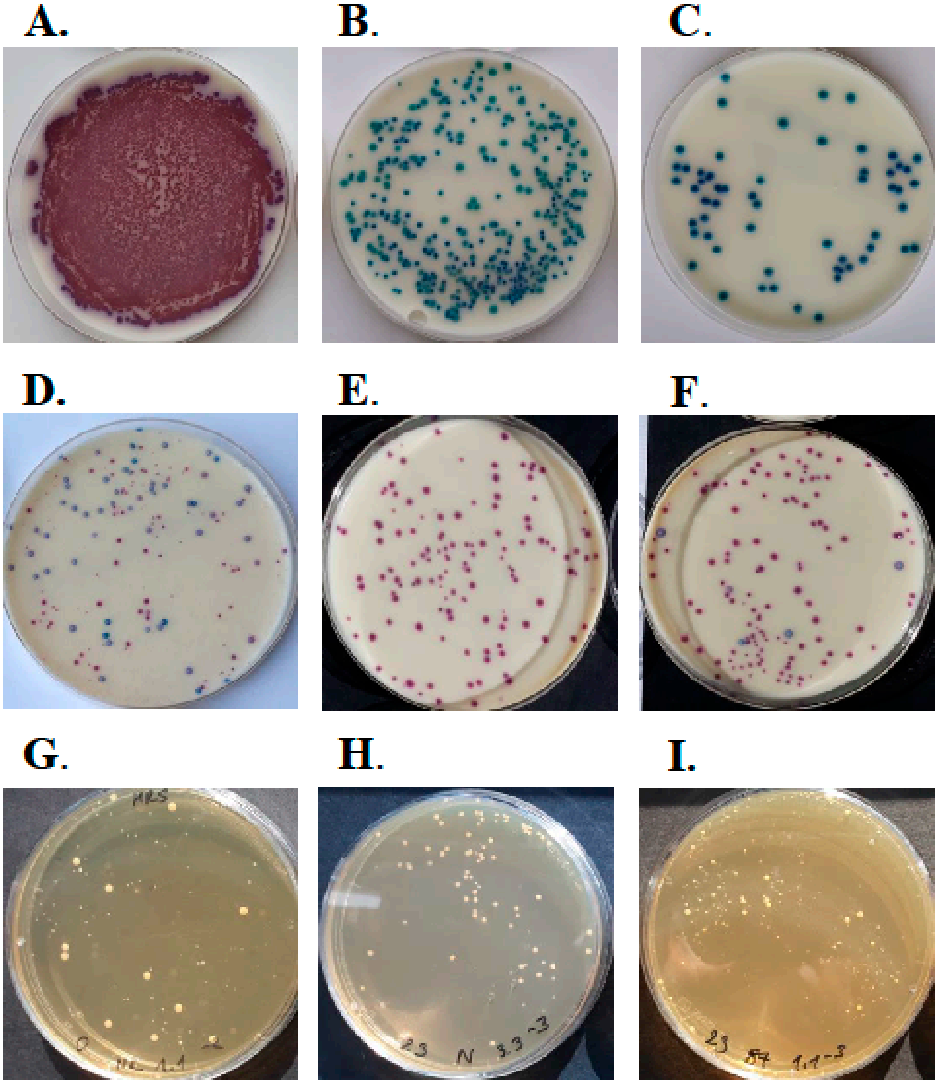

Fig. S1. Demonstrative photos of agar media used in the study. S**1A-C.** RAPID Salmonella agar**. A.** Negative control, day 21 (d: 21, g: NC) **B.** Negative control, day 44 (d: 44, g; NC) **C.** Positive control – group receiving 3% URSM, day 44 (d: 44, g: PC). **S1 D-F.** StrepB agar. **D**. Negative control, day21 (d: 21, g: NC) **E.** Group receiving 3% addition of *B. subtilis* 67Y FRSM, day 44 (d: 44, g: 67) **F.** Positive control – group receiving 3% of URSM, day 44 (d: 44, g: PC). **S1 G-I.** MRS agar. **G.** Negative control, day 21 (d: 21, g: NC) **H.** Positive control – group receiving 3% URSM, day 44 (d: 44, g: PC) **I.** Group receiving 3% *B. subtilis* 87Y FRSM, day 44 (d: 44, g: 87Y).

Table S1. Performance of broiler chickens used in the experiment. NC – negative control; PC – birds receiving 3% addition of URSM; 67 – birds receiving 3% addition of FRSM by *B. subtilis* 67; 87Y – birds receiving 3% addition of FRSM by *B. subtilis* 87Y.

**Day 30**						
**Parameter**	**NC**	**PC**	**67**	**87Y**	**SEM**	**p-value**
Live body weight (g)	Mean 1617.50	Mean 1706.25	Mean 1697.50	Mean 1698.75	38.54	0.859
Daily gains (g)	84.42	75.68	74.89	76.75	3.28	0.760
FCR (kg of feed/kg of gain)	1.58	1.49	1.52	1.49	0.03	0.815
**Day 37**						
**Parameter**	**NC**	**PC**	**67**	**87Y**	**SEM**	**p-value**
Live body weight (g)	Mean 2242.50	Mean 2275.00	Mean 2236.25	Mean 2250.00	36.59	0.987
Daily gains (g)	89.28	81.24	76.95	78.74	5.34	0.881
FCR (kg of feed/kg of gain)	1.60	1.59	1.60	1.60	0.02	0.998
**Day 44**						
**Parameter**	**NC**	**PC**	**67**	**87Y**	**SEM**	**p-value**
Live body weight (g)	Mean 2946.25	Mean 2892.50	Mean 2945.00	Mean 2881.25	51.17	0.963
Daily gains (g)	100.53	88.21	101.24	90.17	7.38	0.909
FCR (kg of feed/kg of gain)	1.57	1.60	1.59	1.61	0.02	0.973

Histophotometric analysis

Table S2. Morphometry of cecal intestines harvested at day 23 of experiment. Numbers represent mean of each parameter. NC – negative control; PC – birds receiving 3% addition of URSM; 67 – birds receiving 3% addition of FRSM by *B. subtilis* 67; 87Y – birds receiving 3% addition of FRSM by *B. subtilis* 87Y.

			Day 44			
**Parameter**	**NC**	**PC**	**67**	**87Y**	**SEM**	**p-value**
**Villus height (µm)**	1750.15	1501.88	1588.22	1628.37	50.61	0.418
**Villus width (µm)**	329.33	230.09	283.84	252.90	20.39	0.278
**Distance between villi (µm)**	42.16	76.34	59.50	68.27	6.13	0.286
**Amount**	6.52	6.62	7.16	6.00	0.39	0.740
